# Molecular docking analysis of selected pyrimidine derivatives with human cyclin-dependent kinase 2

**DOI:** 10.6026/97320630017680

**Published:** 2021-07-31

**Authors:** R Bharathi, N Santhi

**Affiliations:** 1 Research and development centre, Bharathiar University, Coimbatore, 641046, India; 2 Department Of Chemistry, Govt Arts College, C.Mutlur, Chidambaram 608102, India

**Keywords:** Chalcone, pyrimidine, spectral characterization, anti oxidantant activity, DPPH

## Abstract

A series of pyrimidine were synthesized, characterized and evaluated for their antioxidant properties using the human cyclin-dependent kinase-2 protein model. Data shows that the pyrimidine derivatives (compound ID 4G) with para fluoro groups substitution at phenyl ring attached to the 4th position (IC50: 98.5µg/ml), compound 4B bearing hydroxy group at para position of phenyl ring (IC50: 117.8 µg/ml) have significant antioxidant activity. Docking data infer that compounds 4c, 4a, 4h and 4b possess binding energy (-7.9, -7.7, -7.5 and -7.4 kcal.mol-1) with 1HCK (PDB ID) receptor.

## Background:

The development of degenerative diseases like atherosclerosis, ischemic heart disorders, ageing, diabetes mellitus, cancer, and many more is largely attributed to oxidative stress [1]. Oxidative processes lead to the development of free radicals such as superoxid (O2 -), hydroxyl-radical (OH-), and non-free radical (ROS) species such as H2O2 and singlet-oxygen [2]. The oxidation process leads to a reactive oxygen species formation. These reactive species of oxygen cause lipid peroxidation, protein peroxidation, and damage to DNA, and cell degeneration [3]. Throughout the pathology of several diseases, including brain disorders, aggregating platelets, inflammatory diseases, and a variety of other disorders, free radical reactions are therefore involved [4]. Antioxidants can curb free radical chain reactions [5]. In the field of drug design and discovery, therefore, sufficient attention is required when developing effective antioxidants. Phenolic compounds are widespread in plants, protect against the light of UV, insects, bacteria and viruses while preventing competitive plants from developing [6]. Their ability to eliminate hydroxyl radicals, and to help prevent diseases linked to oxidative stress in membranes, proteins and DNA, have traditionally been considered powerful natural antioxidants [7]. Chalcones abound from ferns to higher plants in nature [8]. Aromatic compounds with an unsaturated side chain are frequently in vitro cytotoxic [9]. It was also reported that Chalcones were anti-inflammatory, analgesic and anti pyretia3. Some Chalcones have bactericidal and antifungal activity and some of their derivatives have anti mutagenic activity [10]. Chalcones are 1,3- diphenol-2, propene-1-one, in which a three-carbon α, β- in saturated carbonyl system is connected to two aromatic rings [11]. These are abundant and considered the precursors of flavonoids and iso flavonoids in edible plants. Chalcones have been extensively studied and developed as one of the pharmaceutically significant molecules, both synthetic and natural. Chalcone derivatives have been screened for anti-inflammatory activity [12], chemo phytosis [13], cardiovascular disease [14], anti-cancer activity [15], and cytototoxic activity [16] and anti proliferative activity [17].

Heterocyclic compounds are a class of cyclic organic compounds that are heteroatomically compatible with the carbon framework, such as nitrogen, sulphur, oxygen, etc. Heterocyclic compounds are used in the treatment of a variety of diseases, and possess various pharmacological activities [18]. The heterocyclic ring as the principal structural component for most therapeutic agents used in the current therapy. The heterocyclic rings containing nitrogen are characterised by their easy synthesis, but also by their wide distribution and biological profiles [19]. A literature survey has shown a broad range of bioactives such as antimicrobial, antioxide, anticancer, antimalarial, antidepressant, antihistaminic, antimicrobial and anti-inflammatory, and nitrogen-based heterocycles, i.e. isoxazole and dihydropyrazole [20]. Pyrimidine is a six-member heterocyclic compound that contains two nitrogen atoms at positions 1 and 3. Pyrimidine derivatives have showed various biological activities such as antimicrobial, antitumor, antifungal, and antileishmanial activities and are also useful for the treatment of thyroid and leukemia [21]. Therefore, it is of interest to document the Molecular docking analysis data of selected pyrimidine derivatives with human cyclin-dependent kinase 2 (PDB ID: 1HCK) for further consideration in this context.

## Materials & Methods:

Elemental analysis was carried out using PERKIN ELMER 240 CHN analyzer. All the series of the synthesized product function group were characterized by infrared spectroscopy, which are recorded on SHIMADZU IR spectrometers. The test sample mixed with KBr pellet technique was adopted to record the spectra. 1H and 13C NMR spectrum were recorded on BRUKER AVANCE III AMX-400 spectrometervfor the synthesized compound. The frequency of 400 MHz for the proton NMR spectrum and 100 MHz frequencies for 13C NMR spectrum, CDCl3 and DMSO-D6 used as a solvent.

## Antioxidant activity of synthesized derivatives using DPPH assay:

Hydrogen donating or free radical scavenging capacity of the prepared compounds characterized the in vitro antioxidant potential by 1,1-biphenyl-2-picrylhydrazyl radical (DPPH) method [22]. The antioxidant potential of test sample was measured by estimating the reduction in the absorbance of methanolic solution of DPPH A stock solution of DPPH (33mg in 1L) was prepared using methanol and 5ml of this stock solution was added to 1 ml of test at various concentrations (500, 250, 125, 62.5, 31.5µg/ml). After 60 min, absorbance was measured at 517nm at different conc. (500, 250, 125, 62.5, 31.5µg/ml) in comparison to standard drug. Reference compound used was ascorbic acid and experiment was performed in dark. The scavenging activity was calculated in terms of inhibition employing the following formula:

% Anti-radical activity = [(Control Absorbance-Sample absorbance)/Control absorbance] x100

## Molecular docking:

Chemical structures of all the synthesized compounds were drawn using ChemDraw Ultra 8.0 software. Mol2 files of all the derivatives were converted into .pdb files using Marvin Sketch [23]. All the ligand molecules were allowed to be flexible and their torsional roots were detected and chosen. PDB files were further optimized and converted to pdbqt files for molecular docking by using AutoDock Tools 1.5.6 [24]. The X-ray structure of PDB id: 1HCK was accessible with the help of the protein data bank. Target molecule was download (*.pdb format) and polar hydrogens were added while water molecules were removed by using AutoDock Tools (ADT). Then the *.pdb format of the macromolecule should be converted to *. pdbqt format. Autogrid generation was also performed using AutoDock Tools where values of x, y and z coordinates of active site were determined. Grid based cavity prediction has been attempted for determining binding site.

## Experimental data:

### Synthesis and scheme of (E)-3-(2-amino-3,5-dibromophenyl)-1-(4-substitutedphenyl) prop-2-en-1-one (3a-h):

2-amino-3,5-dibromobenzaldehyde (1) (0.05mol) dissolved in 30 ml of ethanol in 100 ml beaker and The substituted aryl acetophenone (2-9) (0.05mol) were taken and dissolved in 30 ml of ethanol in a 100 ml beaker. The above two solution were taken in a 250 ml RB flask, to this, 2 ml of 30% sodium hydroxide solution was added stirred well until the product formed. Observing on precoated TLC plates identified completion of the reaction. After completion of the reaction, the reaction mixture was poured into crushed ice, and acidified with dil HCl. The product formed were filtered, washed, dried and recrystallized using ethanol. Melting point, elemental analysis, FT-IR, 1H NMR, and 13C NMR spectral studies characterize the newly prepared chalcones 10 - 17.

###  Synthesis and Scheme of 4-(2-amino-3,5-dibromophenyl)-6-(4-substitutedphenyl) pyrimidin-2-amine (4a-h):

About (E)-3-(2-amino-3,5-dibromophenyl)-1-(4-substitutedphenyl) prop-2-en-1-one (0.01 mol), guanidine hydrochloride (0.01 mol),1 ml of 40% sodium hydroxide and 25 ml ethanol added and refluxed for 12 hours continuously in a round bottom 100 ml flask using water condenser. TLC monitored reaction. The resulting solution was poured into ice-cold water and allowed to stand overnight. Precipitate formed were filtered, washed with cold water and dried. The product recrystallized using ethanol. The structure of newly synthesized pyrimidine derivatives 4-(2-amino-3, 5-dibromophenyl)-6-(4-substitutedphenyl) pyrimidin-2-amine are confirmed by FT-IR, 1H NMR and 13C NMR spectral studies.

## Results and Discussion:

### Spectral analysis of compound 3a-h:

The strong absorption stretching frequency band appeared at 1629 - 1699 cm-1 is due to C=O of chalcone moiety and a stretching frequency band strongly appeared at 1551 - 1600 cm-1is due to CH=CH of the chalcone. The stretching frequency band present in the region of 3408-3479 cm-1 is due to N-H bond. Stretching frequency range at 3066 cm-1 is reveals that the presence of aromatic C-H group. The strong singlet appeared at 4.51 - 4.89 ppm is due to ring NH2 proton. The H-α and H-β protons of chalcones occur as two doublets in the ranges 6.96-7.41 ppm (H- α) and 7.61-7.91 ppm (H- β) in the 1H NMR spectra. The other aromatic protons usually appear in between δ 6.97-9.09, depending on the type of aromatic/ heteroaromatic ring and also based on the electronic effects of the substituents present on these rings. The carbonyl carbon of the chalcones usually appears δ 181.9-195.0 in its 13C NMR spectrum. The α-and β- carbon atoms with respect to the carbonyl group give rise to characteristic signals in between δ 121.6-126.4 and δ 135.1-148.0 respectively. Phenyl ring aromatic carbon signals are appeared from 108 to 146.9 ppm.

### Spectral studies of compound 4a-h::

A strong stretching absorption band appeared at 1620 cm-1 is due to C=N of pyrimidine ring. Stretching frequency band present in the region of 3062 cm-1 is due to aromatic C-H protons. A strong stretching absorption band appeared at 3441 cm-1 is due to NH2 of pyrimidine ring. The pyrimidine ring NH2 proton singlet peak appeared at 6.26 ppm. The aromatic protons appeared from 6.37 - 7.93 ppm. The NH2 protons of the phenyl ring appeared 5.32ppm as a singlet. The pyrimidine ring C=N (C (3)), carbon is appeared at 158.9 ppm. Aromatic phenyl ring carbons chemical shift value from 105.1 - 144.3ppm. The pyrimidine ring C-NH2 (C (1)), carbon is appeared at 162.4 ppm.

### Antioxidant activity of chalcone derivatives (4a-h):

Antioxidants are the compounds that protect the cells against the damaging effects of reactive oxygen species, such as singlet oxygen, superoxide, peroxyl radicals, hydroxyl radicals and peroxy nitrite [25]. An imbalance between antioxidants and reactive oxygen species results in oxidative stress, leading to cellular damage. Oxidative stress has been linked to cancer, aging, atherosclerosis, ischemic injury, inflammation and neurodegenerative diseases (Parkinson's and Alzheimer's) [26]. Antioxidant compounds like phenolic acids, polyphenols and flavonoids scavenge free radicals such as peroxide, hydroperoxide or lipid peroxyl and thus inhibit the oxidative mechanisms that lead to degenerative diseases [27]. ROS (reactive oxygen species) is capable of generating free radicals. Mostly it includes hydrogen peroxide (H2O2), superoxide anion (O2•- ) and hydroxyl radical (OH). Chalcone groups play a vital role in trapping the free radicals [28]. Table 1(see PDF) shows the percentage of DPPH radical scavenging activity of pyrimidine derivatives 4a-h.

**Figure 1:**
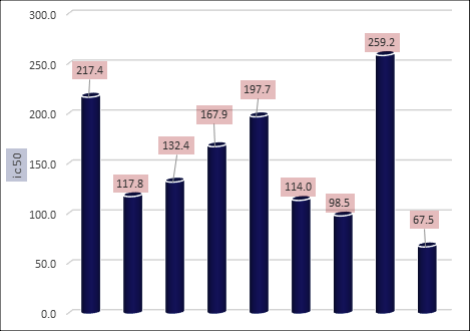
Free radical scavenging assay in percentage for the pyrimidine derivatives 4a-h by DPPH radical scavenging methods.

The synthetic pyrimidine derivatives 4a-h was tested for their free radical scavenging potential. All compounds showed various degrees of radical scavenging activity in DPPH radical scavenging assay, and their IC50 values ranged between 117.8 to 259.2µg/ml. Compound 4g shows better IC50 value (98.5 µg/ml) compared with reference drug ascorbic acid. Derivatives 4b and 4c with IC50 values of 117.8 and 132.4 µg/ml, respectively, showed free radical inhibitory activity that is many folds better than the standard ascorbic acid with IC50 value of 67.5µg/ml, as depicted in Figure 1 and Table 1(see PDF).

Compounds 4b and 4c showed good to moderate activities (Figure 1 & Table 2 - see PDF). The remaining derivatives, including 4a, 4e and 4h showed weak inhibitory activities (Figure 1). A structure-activity relationship established for all compounds that confirmed substitution of various functionalities at the aromatic ring confers free radical scavenging activity to each particular pyrimidine analogue. Analogue 4g, a 4-flurophenyl was found to be the most active pyrimidine among the series, with an IC50 value of 98.5µg/ml, corresponding to 72.18% radical scavenging activity that is as good as 89.36% radical scavenging activity of the standard drug (Table 1 - see PDF). The high activity shown by analogue 4g is due to the position of fluro groups present an aromatic moiety. Thus, the antioxidant activities could be caused by hydrogen atom transfer and/or electron transfer followed by a proton transfer mechanism in a substituent dependent manner.

### Docking study of chalcone derivatives with 1HCK (PDB ID):

**Figure 2:**
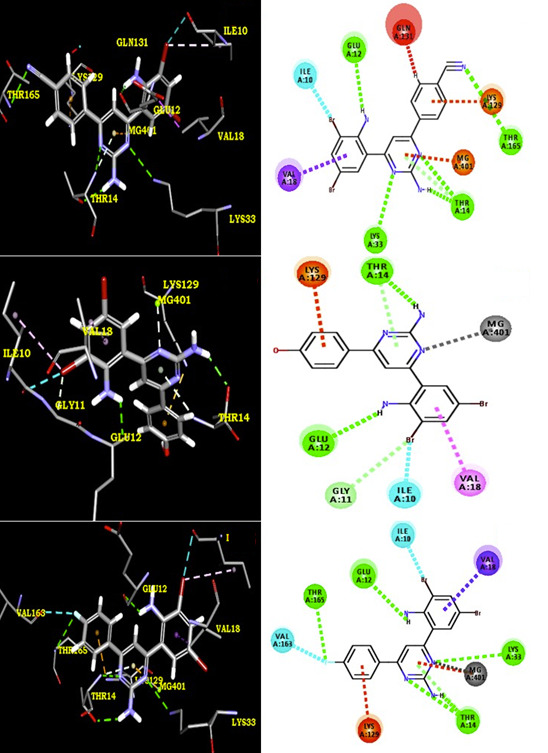
2D & 3D representation of interactions between protein receptor and synthesized derivatives (4a-c)

**Figure 3:**
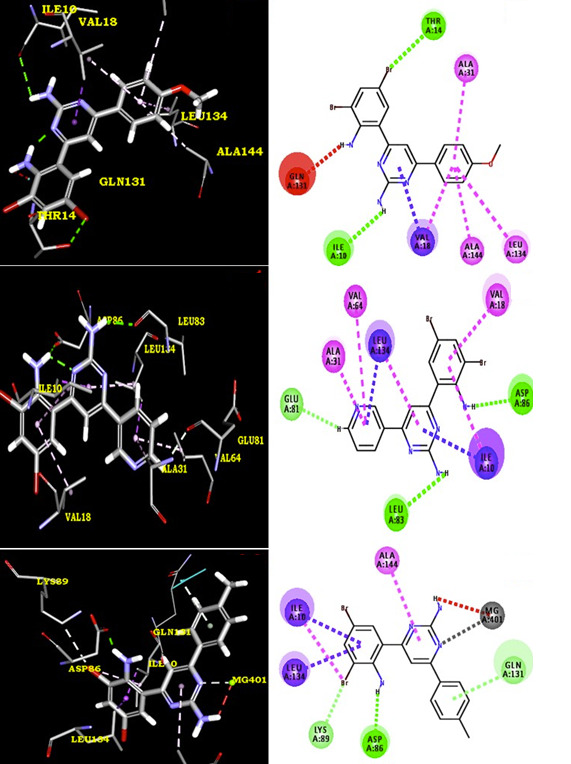
2D & 3D representation of interactions between protein receptor and synthesized derivatives (4d-f)

**Figure 4:**
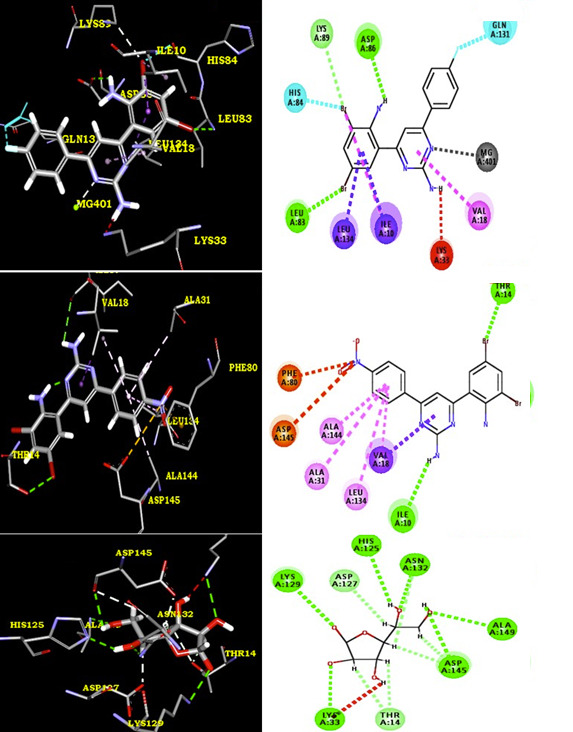
2D & 3D representation of interactions between protein receptor and synthesized derivatives (4g-h & ascorbic acid)

The results of docking studies infer that compounds 4c, 4a, 4h and 4b possess binding energy (-7.9, -7.7, -7.5 and -7.4 kcal.mol-1 ) with 1HCK receptor. Compound 4c form a hydrogen bond with THR 165, GLU 12, LYS 33 and THR 14 of 3LN1 through F ----- H (THR 165), NH2 ----- O (GLU 12),Pyrimidine N----- H(LYS 33),Pyrimidine N----- H(THR 14),Pyrimidine NH2----- O(THR 14). The protein residues of VAL 63, LYS 129, VAL 18 and ILE 10 form alkyl - pi interactions and several VdW and polar / electrostatic interactions. The compound 4a formed a hydrogen bond with LYS 33, THR 14, THR 165 and GLU 12 through Pyrimidine N ----- H (LYS 33), NH2 ----- O (THR 14), Pyrimidine N ----- O(THR 14), CN ----- H (THR 165),NH2 ----- O (GLU 12) . The protein residues of ILE 10, VAL 18, GLA 131 and LYS 129 form alkyl - pi interactions and several VdW and polar / electrostatic interactions were also observed with compound 4a. In addition, compound 4h have a binding energy -7.5 kcal.mol-1. The compound 4h formed a hydrogen bond with THR 14 and ILE 10 through Pyrimidine NH2 ----- O (ILE 10), Br ----- H(THR 14). The residues PHE 80, ASP 145, ALA 144, ALA 31, LEU 134 and VAL 18 have common VdW and polar / electrostatic interactions in compound 4h. The compound 4b formed a hydrogen bond with GLU 12 and THR 14 through NH2 ----- O (GLU 12), pyrimidine NH2 ----- O(THR 14). The protein residues of LYS 129, GLY 11, ILE 10 and VAL 18 form alkyl - pi interactions and several VdW and polar / electrostatic interactions were also observed with compound 4b. Docking confirmation of other compounds like 4b, 4d, 4e and 4f is shown in Figure 2,Figure 3 & Figure 4. We can understand H-bonding donor and acceptor capacity from Table 2 (see PDF).As seen from the results, we concluded that our designed compounds 4c, 4a, 4h and 4b are more active than reference ligand ascorbic acid (-7.9 kcal.mol-1).

## Conclusion:

4-(2-amino-3, 5- dibromophenyl)-6-(4-substitutedphenyl) pyrimidin-2-amine (4a-h) have been synthesized by treating guanidine hydrochloride with various substituted chalcones (E)-3-(2-amino-3,5-dibromophenyl)-1-(4-substitutedphenyl) prop-2-en-1-one (3a-h) in ethanol solvent. Biological activities of the synthesized compounds were evaluated by antioxidant activity. The methyl and fluoro substituted compounds 4f and 4g show better activity in antioxidant activity. All the ligand inhibition activity was measured by molecular docking studies. On the basis of molecular docking studies compounds 4c (chloro) and 4a (cyano) have maximum binding score in the case of 1HCK receptor. Finally, Compounds having electron withdrawing substituent at 4th position of phenyl ring shows better biological activity than another substituent.
